# IRGM in autophagy and viral infections

**DOI:** 10.3389/fimmu.2012.00426

**Published:** 2013-01-17

**Authors:** Denitsa S. Petkova, Christophe Viret, Mathias Faure

**Affiliations:** Laboratory of Autophagy, Infections and Immunity, INSERM, U1111, CIRI, Université Lyon 1Lyon, France

**Keywords:** autophagy, IRGM, virus, infection, immunity, interferon

## Abstract

Autophagy is a cell autonomous process allowing each individual cell to fight intracellular pathogens. Autophagy can destroy pathogens within the cytosol, and can elicit innate and adaptive immune responses against microorganisms. Nevertheless, numerous pathogens have developed molecular strategies enabling them to avoid or even exploit autophagy for their own benefit. IRGM (immunity-related GTPase family M) is a human protein recently highlighted for its contribution to autophagy upon infections. The physical association of IRGM with mitochondria and different autophagy-regulating proteins, ATG5, ATG10, SH3GLB1, and LC3, contribute to explain how IRGM could regulate autophagy. Whereas IRGM is involved in autophagy-mediated immunity against bacteria, certain viruses seem to have developed strategies to manipulate autophagy through the selective targeting of this protein. Furthermore, *irgm* variants are linked to infection-associated human pathologies such as the inflammatory Crohn’s disease. Here, we discuss how IRGM might contribute to human autophagy upon viral infection, and why its targeting might be beneficial to virus replication.

## INTRODUCTION

**FIGURE 1:**
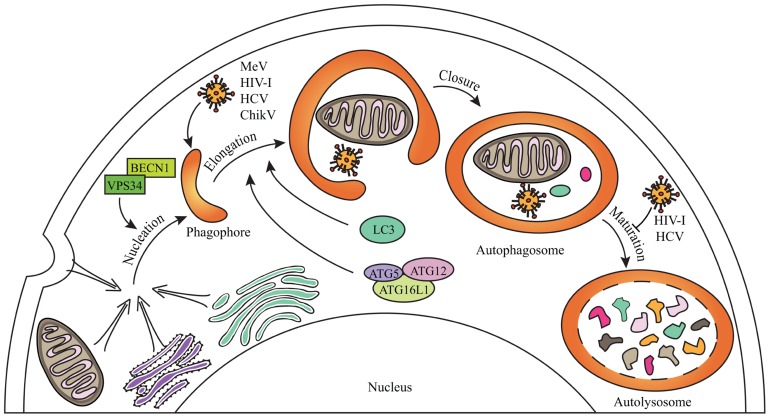
**General steps of mammalian autophagy**. Autophagy engulfs portions of the cytosol through three main steps. The nucleation induces the formation of an isolation membrane which could emerge from different membrane sources (the endoplasmic reticulum, the Golgi apparatus, the plasma membrane, the mitochondria) to form a phagophore which elongates to form double-membraned autophagosome vesicles. Autophagosome can sequester cytosolic material including senescent organelles such as mitochondria, long-lived proteins or intracellular pathogens, through independent selective autophagies (for schematic simplification, all were represented within one single autophagosome). Autophagosome ultimately fuses with lysosomes during the maturation step to form autolysosomes where degradation occurs. Some of the crucial proteins involved in the different phases of autophagy are indicated. Several viruses can induce a complete autophagy flux, as MeV or ChikV, whereas others can inhibit autophagosome maturation, as HIV-1 and HCV, in order to improve their replication (see text for details).

As obligatory intracellular parasites, viruses are continually faced with the degradative mechanism of macroautophagy (thereafter referred to as autophagy; **Figure 1**). Autophagy can destroy infectious virions or virus components that are essential for replication (Levine, 2005; Richetta and Faure, 2012). Furthermore, autophagy can deliver viral genomes to TLR-containing endosomes, which sets off synthesis of antiviral type I interferon (IFN-I; Lee et al., 2007). Autophagy can also contribute to virus-derived peptide presentation on class I and class II major histocompatibility complex (MHC) molecules to trigger antiviral CD8^+^ and CD4^+^ T cells responses, respectively (English et al., 2009; Munz, 2009). Thus, autophagy is an intrinsic cellular antiviral process able to enhance innate responses and to link them to adaptive immunity to optimize the fight against viruses.

Numerous viruses have developed molecular strategies to counteract autophagy. Certain viruses developed properties enabling them to inhibit the autophagy flux. For instance, herpes simplex virus (HSV)-1 or the cytomegalovirus (CMV) can inhibit autophagy induction by targeting BECLIN1 (BECN1), an essential autophagy-associated protein, through interactions with viral proteins (Orvedahl et al., 2007; Chaumorcel et al., 2012). Other viruses avoid autophagy degradation by inhibiting autophagosome maturation such as human immunodeficiency virus (HIV)-1 and influenza A virus which prevent autophagosome maturation through the physical interaction of one viral protein with BECN1 (Gannage et al., 2009; Kyei et al., 2009). Finally, viruses can induce a complete productive autophagy process and exploit it as a source for metabolites or as a platform for improving their own replication (Jackson et al., 2005; Heaton and Randall, 2010). Thus, host-virus co-evolution may have led to the selection of very different mechanisms used by viruses to avoid or exploit autophagy.

The understanding of the molecular pathways linked to the proviral or antiviral functions of autophagy is still at its beginning, especially regarding the molecular interplay between viruses and autophagy proteins. The human immunity-related GTPase family M (IRGM) protein was shown to be widely targeted by RNA viruses, several among which can exploit autophagy in human cells to improve their replication (Gregoire et al., 2011,^2012^). However, how IRGM regulates autophagy upon infections remains unknown. This aspect might be of great interest in several human pathologies for which *irgm* was recently found to be involved.

## IRGM AND AUTOPHAGY IN INFECTIONS

Unlike its mouse ortholog, the human *irgm* gene expression is not under the control of IFN-γ (Bekpen et al., 2005). Whereas 23 immune-related genes (*irg*) genes exist in mice and play immune-related functions, the IRGM-mediated cell autonomous resistance mechanisms have been first lost in primates due to deleterious mutations. However, *irgm* gene function was restored in some primates including *Homo sapiens*, possibly due to the insertion of a retroviral element that promotes its expression (Bekpen et al., 2009). In human, five different IRGM splice isoforms that differ in their C-terminal ends could be expressed, but their individual endogenous expression has not yet been documented (Bekpen et al., 2005; Singh et al., 2010); endogenous IRGM as well as overexpressed IRGMd isoform can localize in mitochondria (Singh et al., 2010; Gregoire et al., 2011).

One of the first molecular demonstrations of a role of autophagy in immune responses against intracellular microorganisms, involved the murine ortholog of IRGM, IRGM1 (Gutierrez et al., 2004), previously described to be involved in the vacuolar trafficking of phagosomes containing *Mycobacterium tuberculosis* (MacMicking et al., 2003). *M. tuberculosis* entrapped within phagosomes can avoid its destruction by preventing phagosome fusion with lysosomes but the rerouting of *M. tuberculosis*-containing phagosomes to the autophagic machinery can ultimately degrade the bacteria. The treatment of murine macrophages with IFN-γ induces autophagy *via* IRGM1 and protects from *M. tuberculosis* (Gutierrez et al., 2004; Singh et al., 2006). IRGM-mediated autophagy also contributes to protection in human cells against intracellular *M. tuberculosis*, *Escherichia coli*, and* Salmonella typhimurium* (Singh et al., 2006; McCarroll et al., 2008; Lapaquette et al., 2010; Brest et al., 2011). The fact that there appears to be no role for IRGM or for its murine ortholog IRGM1 in the regulation of autophagy in absence of infection suggests a pathogen-specific function in autophagy for these proteins (Gregoire et al., 2011; Matsuzawa et al., 2012).

## IRGM IN VIRUS-MEDIATED AUTOPHAGY

### IRGM IS A COMMON TARGET OF RNA VIRUSES

RNA viruses genome encodes very few proteins including non-structural proteins that are often dedicated to prevent antiviral responses (Katze et al., 2008). To optimize replication, individual viral proteins could target several different host-cell proteins to counteract cellular antiviral responses. Alternatively, viral proteins could be dedicated to the efficient targeting of few host-cell proteins to counteract essential biological functions. The analysis of the interactions between 44 autophagy-associated human proteins and 83 viral proteins belonging to different RNA virus families revealed IRGM as the most targeted autophagy-associated protein by these viruses. IRGM can interact with 12 viral proteins belonging to five different viruses, Chikungunya virus (ChikV), Mumps virus (MuV), Hepatitis C virus (HCV), Measles virus (MeV), and HIV-1 (Gregoire et al., 2011). Except for MuV for whom no autophagy-related studies were yet reported, all other viruses manipulate autophagy.

### IRGM AND VIRUSES EXPLOITING THE AUTOPHAGY FLUX

Measles virus infection increases the formation of *de novo* autophagosomes by inducing the autophagy flux (**Figure 1**; Joubert et al., 2009; Meiffren et al., 2010; Gregoire et al., 2011). Genetic inhibition of autophagy limits MeV viral particles production, indicating that MeV exploits autophagy to replicate. The reduced expression of cellular IRGM with specific siRNA decreased MeV replication in HeLa cells. Furthermore, the non-structural MeV-C protein can interact with IRGM, and its single overexpression induces autophagy through an IRGM-dependent pathway (Gregoire et al., 2011). Thus, in the course of MeV infection, the MeV-C/IRGM interaction might contribute to the exploitation of autophagy by MeV.

Chikungunya virus infection also induces the autophagy flux (**Figure 1**; Krejbich-Trotot et al., 2011; Joubert et al., 2012). Whereas inhibition of autophagy limits ChikV viral particles production, the experimental promotion of autophagy improved its replication (Krejbich-Trotot et al., 2011). ChikV replication is required to induce autophagy upon infection, and, as a consequence, autophagy delays cell death, which limits ChikV-associated pathogenesis, but favors its dissemination (Joubert et al., 2012). ChikV infection induces endoplasmic reticulum and oxidative stresses that independently can trigger autophagy (Joubert et al., 2012). However, it is unknown whether ChikV proteins contribute directly to autophagy induction and/or maintenance in infected cells. Especially, IRGM was found to interact with ChikV-NS2 and E3 proteins (Gregoire et al., 2011). It will be interesting to determine whether ChikV/IRGM interaction contributes to autophagy manipulation.

### IRGM AND VIRUSES INHIBITING AUTOPHAGY MATURATION

During HIV-1 infection, autophagy manipulation strategies depend on the type of infected cells. The exposition of HIV-1-ENV protein on membranes of infected cells induces autophagy in uninfected CD4^+^ T cells leading to their apoptotic cell death (Espert et al., 2006). However, HIV-1 inhibits autophagy in infected CD4^+^ T cells, which facilitates replication (Espert et al., 2009). In dendritic cells (DC), HIV-1 inhibits autophagy through exhaustion of the mTOR signaling pathway (Blanchet et al., 2010). However, autophagy is induced in HIV-1-infected macrophages where HIV-1–NEF protein can interact with BECN1 to inhibit the maturation of autophagosomes, what is required for an efficient replication of HIV-1 (Espert et al., 2009; Kyei et al., 2009). NEF can also interact with IRGM and the overexpression of NEF induces an IRGM-dependent accumulation of autophagosomes (Kyei et al., 2009; Gregoire et al., 2011). Thus, while NEF–BECN1 interaction could prevent autophagosome maturation, NEF–IRGM interaction could be involved in autophagy induction upon HIV-1 infection in macrophages. Through its interaction with distinct autophagy-associated proteins, a unique HIV-1 protein could finely regulate autophagy. Interestingly, a NEF deficient strain of HIV-1 does not induce autophagosome accumulation, suggesting indeed that, besides preventing autophagosome maturation, NEF is involved in the induction of autophagy by HIV-1 (Kyei et al., 2009).

Infection by HCV also induces autophagy. This induction is independent of mTOR (Su et al., 2011; Shrivastava et al., 2012), and the contribution of the unfolded protein response remains unclear (Sir et al., 2008; Mohl et al., 2012). However, autophagy is required for an optimal HCV replication since inhibition of autophagy affects HCV replication (Dreux et al., 2009; Tanida et al., 2009; Gregoire et al., 2011). Reports have shown that HCV infection could either induce a complete autophagy flux or inhibit autophagosome maturation. This discrepancy might result from the models used and/or the kinetics of infection. HCV infection was shown to prevent autophagosome maturation at an early time of infection (Sir et al., 2008; Gregoire et al., 2011; Su et al., 2011). At a later one a complete autophagy flux was reported (Ke and Chen, 2011; Mohl et al., 2012). Interestingly, a subgenomic replicon expressing the non-structural NS3-5B proteins induces autophagy (Mohl et al., 2012). Furthermore, IRGM can interact with HCV-NS3, and the reduced expression of IRGM prevents HCV-induced and HCV-NS3-induced autophagy, and limits HCV replication (Gregoire et al., 2011).

Thus, viruses that manipulate autophagy either by benefiting from the complete autophagy flux or by inhibiting the maturation step, target IRGM. Beyond its role in virus biology, how IRGM contributes to the orchestration of autophagy upon viral infection remains to be understood.

## IRGM IN AUTOPHAGY INDUCTION UPON VIRAL INFECTIONS

### IRGM AND AUTOPHAGY-ASSOCIATED PROTEINS

**FIGURE 2:**
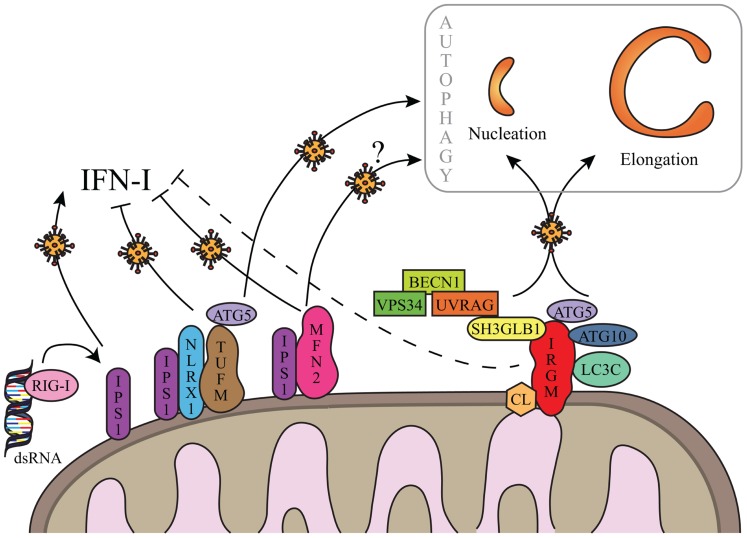
**Hypothetical regulation of autophagy by IRGM upon viral infections**. IRGM is associated to mitochondria via cardiolipin (CL). Upon viral infection, IRGM could interact with four different autophagy-associated proteins which could support autophagosome biogenesis from mitochondria membrane, by regulating nucleation and/or elongation steps of autophagy. IRGM-mediated autophagy might correlate to a decrease of IFN-I synthesis similarly to what was described for TUFM and MFN2. Both processes could benefit to viruses to improve their replication. Note that MFN2-mediated autophagy has not yet been reported in the context of viral infection (see text for details). As represented, viral double-stranded (ds)RNA can be recognized by RIG-I. IFN-I inhibition via IRGM is not yet demonstrated (dashed line) and putative not mitochondrial IRGM isoforms are not represented (see text for details).

To date, only four cellular proteins were identified to interact with IRGM: ATG5, ATG10, MAP1LC3C, and SH3GLB1 (**Figure 2**). All these proteins contribute to autophagy, supporting the idea that IRGM plays an essential role in this process (Gregoire et al., 2011). ATG10, a conjugating E2-like protein, contributes to the assembly of the ATG12/ATG5 complex that binds ATG16L1 to form macromolecular ATG12/ATG5/ATG16L1 complexes essential for the elongation of the phagophore (**Figure 1**; Xie and Klionsky, 2007). MAP1LC3C is a member of the MAP1LC3 (known as LC3) sub-family and is also required for elongation of the phagophore through lipidation with phosphatidylethanolamine and anchoring within the extending phagophore (**Figure 1**; Weidberg et al., 2010). Finally, SH3GLB1 (also known as Bif-1) is a positive regulator of the nucleation process that initiates autophagosome formation, *via* its interaction with UVRAG, a protein of the BECN1/VPS34 complex. In nutrient deprived cells, SH3GLB1 colocalizes with ATG5 and LC3 to the autophagosome and potentiates the activation of the class III PI(3)-kinase VPS34 to promote autophagosome biogenesis (**Figure 2**; Takahashi et al., 2007). Thus, all the proteins known to interact with IRGM regulate one of the initial steps of autophagosome biogenesis, suggesting that IRGM might contribute to the nucleation and/or the elongation of autophagic vesicles through its interaction with one or several of these proteins. These interactions could be facilitated upon viral infection (**Figure 2**). Through the dampening of antiviral IFN-I synthesis, this targeting might be of further benefit to viruses as discussed below.

### IRGM, MITOCHONDRIA, AUTOPHAGY, AND IFN-I IN VIRAL INFECTIONS

Overexpressed GFP-fused IRGMd was suggested to be translocated to the inner membrane of mitochondria *via* its association with cardiolipin (CL; Singh et al., 2010). CL is a phospholipid abundant in the inner mitochondrial membrane that is however also found in the outer membrane and at the contact sites between the two membranes (Schlame et al., 2000; Schug and Gottlieb, 2009). Thus IRGMd might also be associated to CL linked to the outer membrane of mitochondria, and exposed to the cytosol. The four other overexpressed GFP-fused IRGM isoforms (IRGMa/b/c/e) were not found associated to mitochondria (Singh et al., 2010). However, endogenous IRGM expression, detected with an antibody with putative ability to recognize all IRGM isoforms, is found associated to mitochondria suggesting that: (i) all endogenous IRGM can be located to mitochondria, (ii) IRGMd is the most expressed isoform, or (iii) due to possibly distinct folding among the isoforms, the epitope for the antibody is only accessible on IRGMd (**Figure 2**; Singh et al., 2010; Gregoire et al., 2011). Interestingly, among the proteins interacting with IRGM at least two were found partially associated with mitochondria, SH3GLB1 and ATG5. A fraction of SH3GLB1 localizes to mitochondria where it may contribute to the regulation of morphological dynamics of the outer mitochondrial membrane (Karbowski et al., 2004), and to mitochondria-dependent apoptotic signals by interacting with the proapoptotic protein BAX (Takahashi et al., 2005). ATG5 was also shown to associate with mitochondria through its binding to IPS-1, a mitochondria-associated adaptor which relays signals from viral genome-detecting cytosolic receptors RIG-I and MDA5, in order to promote IFN-I synthesis. This interaction contributes to the down-regulation of IFN-I production during viral infection (Jounai et al., 2007). A possible hypothesis for the molecular contribution of IRGM in autophagy would be that IRGM interacts/recruits its protein partners at the mitochondria to induce autophagy upon infections especially as mitochondria is one possible source of membrane for autophagosome biogenesis (**Figures 1 and 2**; Hailey et al., 2010). Furthermore, the IRGM targeting to mitochondria could allow viruses to limit IFN-I production similarly to two other mitochondrial proteins, MFN2 and TUFM, which were shown to dampen IFN-I production while inducing autophagy (**Figure 2**). Indeed, MFN2 contributes to the supply of mitochondria membranes for the biogenesis of autophagosome (Hailey et al., 2010), and can down-regulate the production of IFN-I upon viral infection by interacting with IPS-1 (Yasukawa et al., 2009). Similarly, TUFM recruits the ATG5/ATG12 complex in order to induce autophagy, while it prevents RIG-1/IPS-1 signal transduction for IFN-I production *via* its interaction with NLRX1 (Lei et al., 2012). The antagonistic activities of TUFM and MFN2 on autophagy and IFN-I production were shown to benefit to virus replication (Yasukawa et al., 2009; Lei et al., 2012). As IRGM is associated to mitochondria and modulates autophagy induction upon virus infection, it would be important to evaluate its contribution to the ability of viruses to dampen IFN-I production; all the viruses described to date to target IRGM are known to inhibit IFN-I production. Thus, different mitochondrial proteins, including IRGM, might have dual functions upon virus infection, by inducing autophagy ultimately exploited by viruses, while restricting the innate antiviral response; the selective targeting of these proteins would offer an evident advantage for infectious viruses to replicate within a cell. It remains however possible that cytosolic isoforms of IRGM contributes to autophagy induction upon viral infection.

### IRGM AND AUTOPHAGY-MEDIATED VIRUS-DERIVED PEPTIDE MHC LOADING

It recently became clear that autophagy which is constitutively active in antigen-presenting cells (APCs), can regulate adaptive immune responses by promoting the access of antigens from intracellular pathogens to compartments that assemble peptide:MHC class II complexes for presentation to CD4^+^ T cells (Munz, 2009). Among viruses targeting IRGM, it was observed that the response of HIV-1 gag-specific CD4^+^ T cells to DC that process the virus was drastically reduced upon either pharmacological or genetic inhibition of autophagy, indicating a deficient capacity to process and present MHC class II-restricted HIV-1 determinants when autophagy is impaired (Blanchet et al., 2010). The negative regulation of autophagy in DCs by HIV-1 could thus help the virus evade CD4^+^ T cell responses. As to presentation by MHC class I molecules, it is known that in mouse DCs, IRGM3/IGTP (another murine ortholog or IRGM) plays an important role in cross-presentation of phagocytosed protein antigens to conventional CD8^+^ T cells without impacting antigen presentation to CD4^+^ T cells (Bougneres et al., 2009).

## *irgm* VARIANTS IN HUMAN PATHOLOGIES AND VIRAL INFECTIONS

Recent studies identified *irgm* variants as susceptibility genes for Crohn’s disease (CD), tuberculosis (TB), gastric cancer and autoimmune systemic lupus erythematosus (SLE).

### IRGM, CD, AND VIRAL INFECTION

Crohn’s disease is a chronic inflammatory bowel disease resulting from an aberrant immune response toward the intestinal flora that leads to inflammation and tissue damages (Xavier et al., 2008). Genome-wide association studies identified polymorphisms in two autophagy-associated genes, *atg16L1* and *irgm*, that are linked to CD. CD-associated *irgm* polymorphisms, that influence or not the primary protein sequence, were both reported (Parkes et al., 2007; McCarroll et al., 2008; Moon et al., 2012). Interestingly, the gut mucosa of CD patients harbors an increased amount of the pathogenic Adherent-invasive *E. coli* (AIEC) and IRGM-dependent autophagy contributes to fight pathogenic AIEC (Lapaquette et al., 2012). Moreover, microRNA (miR)-196 binds strongly the *irgm* protective haplotype, whereas expression of the risk haplotype remains intact thus leading to overall deregulation of IRGM expression (Brest et al., 2011). miR-196 was found overexpressed in inflamed ileum and colon of patients, independently of the protective or risk *irgm* haplotype. As a result, IRGM was less expressed in individuals with the protective genotype. Furthermore, the transfection of HEK293T cells with miR-196 resulted in a decreased autophagy flux, indicating that miR-196 acts as a negative regulator of autophagy *via* IRGM upon AIEC infection. These studies suggested that the cornerstone of autophagy regulation by IRGM upon infection could be its fine tuned level of expression.

Interestingly, a viral infection-plus-susceptibility autophagy gene interaction could contribute to the onset of CD. Indeed, the hypomorphic expression of *atg16l1* develops a CD-like pathology in mice only upon infection with a viral strain of murine norovirus (Cadwell et al., 2010). This study pointed toward a genotype-specific viral trigger of a pathology very similar to CD. It would be interesting to investigate the role of viral infections in *irgm* variant expressing CD patients, for a possible contribution of virus/IRGM interactions in the onset or the development of CD.

### IRGM IN TB, GASTRIC CANCER, AND SLE

As mentioned above, IRGM contributes to the control of *M. tuberculosis* in macrophages *via* autophagy. Interestingly, an *irgm* polymorphism protects from TB caused by Euro-American subgroups of *M. tuberculosis* (Intemann et al., 2009). It was proposed that when the polymorphism occurs, IRGM is more expressed resulting in enhanced autophagy and explaining a more efficient destruction of bacteria. Conversely, several different polymorphisms in the *irgm* gene have been found to result in an increased susceptibility to TB in Chinese and African American populations (Che et al., 2010; King et al., 2011). In African American populations one CD-related polymorphism was associated positively with TB suggesting a possible link between CD and an infectious etiology. *Irgm* polymorphism is possibly also a risk factor for gastric cancer (Burada et al., 2012). Although deregulation of autophagy is well established to be associated with cancer (White, 2012), a role for IRGM in these diseases has to be further determined. Similarly, a genetic-association study suggested that *irgm* variants are linked to SLE, an autoimmune disease (Zhou et al., 2011). A role of IRGM in these diseases remains to be fully depicted as well as a potential influence of viral infections on such role.

## CONCLUSION

In an infected cell a virus has to counteract cell autonomous defense mechanisms while exploiting elementary cellular processes to replicate efficiently. By selectively targeting autophagy, viruses might accomplish both. As discussed here, IRGM could be a key protein for autophagy manipulation upon viral infection. The molecular organization involving IRGM in autophagy during viral infections requires further investigations. While interactions of IRGM with its protein partners were only observed in transfected cells for the time being, it would be important to visualize these interactions between endogenous proteins and during productive infections. It would also be crucial to understand why and how IRGM plays an antibacterial function, whereas it seems to act as a proviral factor. Furthermore, the role of IRGM could be cell type-specific and, as described for several autophagy-related proteins, IRGM might have non-autophagy-related functions upon infections. In regards of the link of numerous *irgm* variants with human pathologies, the comprehension of the role(s) of IRGM in autophagy-mediated immunity could be of crucial importance to fight infectious viruses and human pathologies.

## Conflict of Interest Statement

The authors declare that the research was conducted in the absence of any commercial or financial relationships that could be construed as a potential conflict of interest.
